# Utilizing plasma-generated N_2_O_5_ gas from atmospheric air as a novel gaseous nitrogen source for plants

**DOI:** 10.1007/s11103-024-01438-9

**Published:** 2024-04-08

**Authors:** Taro Yamanashi, Shouki Takeshi, Shota Sasaki, Keisuke Takashima, Toshiro Kaneko, Yasuhiro Ishimaru, Nobuyuki Uozumi

**Affiliations:** 1 Department of Biomolecular Engineering, Graduate School of Engineering, Tohoku University, Aobayama 6-6-07, Sendai, 980-8579 Japan; 2 Department of Electronic Engineering, Graduate School of Engineering, Tohoku University, Aobayama 6-6-05, Sendai, 980-8579 Japan

**Keywords:** Nitrogen, Plasma device, N_2_O_5_ gas, Gas fertilizer, Essential nutrients, Sustainability

## Abstract

Fixing atmospheric nitrogen for use as fertilizer is a crucial process in promoting plant growth and enhancing crop yields in agricultural production. Currently, the chemical production of nitrogen fertilizer from atmospheric N_2_ relies on the energy-intensive Haber–Bosch process. Therefore, developing a low-cost and easily applicable method for fixing nitrogen from the air would provide a beneficial alternative. In this study, we tested the utilization of dinitrogen pentoxide (N_2_O_5_) gas, generated from oxygen and nitrogen present in ambient air with the help of a portable plasma device, as a nitrogen source for the model plant *Arabidopsis thaliana*. Nitrogen-deficient plants supplied with medium treated with N_2_O_5_, were able to overcome nitrogen deficiency, similar to those provided with medium containing a conventional nitrogen source. However, prolonged direct exposure of plants to N_2_O_5_ gas adversely affected their growth. Short-time exposure of plants to N_2_O_5_ gas mitigated its toxicity and was able to support growth. Moreover, when the exposure of N_2_O_5_ and the contact with plants were physically separated, plants cultured under nitrogen deficiency were able to grow. This study shows that N_2_O_5_ gas generated from atmospheric nitrogen can be used as an effective nutrient for plants, indicating its potential to serve as an alternative nitrogen fertilization method for promoting plant growth.

**Supplementary Information:**

The online version contains supplementary material available at 10.1007/s11103-024-01438-9.

## Key message

We identified plasma-generated N_2_O_5_ gas as a promising nitrogen fertilizer with the capacity to promote agricultural production by utilizing atmospheric nitrogen.

**Supplementary Information:**

The online version contains supplementary material available at 10.1007/s11103-024-01438-9.

## Introduction

Nitrogen is an essential element for plant growth, and the availability of nitrogen directly impacts crop yield (Cui et al. 2018; Luo et al. 2020). Plants cannot directly utilize atmospheric nitrogen as a nitrogen source, and therefore they rely on nitrogen sources that have undergone nitrogen fixation, converting atmospheric nitrogen into a form that plants can absorb (Charles et al. 2010; Pikaar et al. 2017). The Haber–Bosch process has significantly increased the supply of nitrogen for crop production, leading to higher crop yields and supporting a larger population. Nitrogen fixation through this process surpasses the amounts of nitrogen fixed by living organisms (Pikaar et al. 2017). As the global population continues to grow, the demand for nitrogen fertilizers rises and will reach to 150 Mton by 2050 (Pikaar et al. 2022). However, the Haber–Bosch method has several drawbacks. Its production requires approximately 2% of the world’s annual energy consumption and emits over 300 million tons of CO_2_ due to the chemical conversion of highly stable N_2_ molecules through reactions at high temperatures and pressures (350–550 °C, 150–350 atm) (Patil et al. 2015). The requirement for large-scale facilities leads to high expenses and the need for construction sites. As a result, nitrogen fertilizers currently represent the largest cost factor in agriculture in industrialized countries and their use is becoming increasingly difficult in developing countries. Furthermore, a significant portion (50–75%) of the supplied nitrogen is not absorbed by plants and is instead lost through leaching into the soil. The nitrogen forms that remain in the soil are a cause of excessive accumulation in soil, which affects plant growth adversely (Liu et al. 2020). Consequently, there is a growing need to explore new, sustainable, and environmentally friendly alternatives to the Haber–Bosch method of nitrogen fixation and nitrogen supplementation (Cherkasov et al. 2015; Fradgley et al. 2021; Chen et al. 2021).

Plasma-based nitrogen fixation is gaining attention as an alternative procedure to supply nitrogen (Patil et al. 2015; Li et al. 2018). Plasma is a highly energetic state of matter that can generate nitrogen oxides by dissociating nitrogen and oxygen molecules present in air (Graves 2012; Ochi et al. 2017). This process can be performed under relatively moderate reaction conditions, such as atmospheric pressure and ambient temperature, and requires minimal energy consumption and start-up time to initiate the process which ensures compatibility with renewable energy sources. Therefore, plasma-based nitrogen fixation is being widely investigated as a promising approach for establishing sustainable agricultural systems (Graves 2012; Patil et al. 2015; Puač et al. 2018; Gao et al. 2022). Traditional plasma-based nitrogen fixation has been employed for the synthesis of NH_3_ and NOx (Bogaerts and Neyts 2018). More recently, a plasma device has been developed to generate dinitrogen pentoxide (N_2_O_5_) from atmospheric nitrogen and oxygen (Sasaki et al. 2021). N_2_O_5_ is a highly reactive compound that decomposes at high temperatures and is hygroscopic, readily reacting with water and rapidly converting into two nitrate ions (NO_3_^−^). This knowledge inspired us to investigate whether plasma-generated N_2_O_5_ gas could function as a nitrogen source for plant culture, i.e. an alternative form of nitrogen fixation.

In this study, we evaluated the potential of plasma-generated N_2_O_5_ gas as a nitrogen fertilizer. Addition of N_2_O_5_ gas to growth medium supported growth of plants cultured in otherwise nitrogen-deficient medium, however direct and prolonged exposure of plants to the gas itself inhibited growth. To overcome this problem, we successfully tested several alternative approaches for supplying plasma-generated N_2_O_5_ gas to the plants. We conclude that plasma-generation of N_2_O_5_ has potential to serve as a novel nitrogen fixation procedure that will allow the use of atmospheric nitrogen in agricultural production.

## Methods

### Plant growth conditions

In this research, a series of experiments were carried out using the model plant *Arabidopsis thaliana*. Plants were grown in a growth chamber (LPH-410S; Nippon Medical & Chemical Instruments Co. Ltd.) set to 16 h light, 8 h dark, 100–120 mmol of photons m^−2^ s^−1^ with daylight LEDs, and 22 °C, on vermiculite as substrate. To support growth, each plant was supplied with 100 ml/week of liquid medium by placing the pot holding the plants and vermiculite into a larger container containing the medium. Nitrate sufficient medium (+N medium) was composed of 1.4 M CaNO_3_, 0.35 M MgSO_4_–7H_2_O, 2 M KCl diluted 1000-fold in ultrapure water and 100 mM Fe[III]–EDTA diluted 10,000-fold. Micronutrients (70 mM H_3_BO_3_, 20 mM MnCl_2_–4H_2_O, 0.3 mM CuSO_4_–4H_2_O, 1 mM ZnSO_4_–7H_2_O, 0.2 mM K_2_MoO_4_, 0.1 mM CoCl_2_–6H_2_O) were prepared in 1000-fold dilutions. Nitrate-free medium (–N medium) was prepared in ultrapure water with 1.4 M CaCl_2_, 0.35 M MgSO_4_–7H_2_O, 2 M KCl diluted 1000-fold and 100 mM Fe[III]–EDTA diluted 10,000-fold. Micronutrients were prepared in a 1000-fold dilution.

### Plasma gas generation with portable plasma N_2_O_5_ device

A portable plasma N_2_O_5_ generation device was used as previously described to produce N_2_O_5_ from air (Sasaki et al. 2021). N_2_O_5_ gas generated from dried air contained approximately 310 ppm of N_2_O_5_ and was delivered at a flow rate of 2 L/min. When the dry N_2_O_5_ gas (2 L/min) was mixed with humidified air (0.2 L/min), N_2_O_5_ gas was converted to HNO_3_ gas during a 21 s-reaction process in a perfluoroalkoxy alkanes tube (10-m length and 10-mm inner diameter). N_2_O_5_ gas and HNO_3_ gas were flown through a gas cell with 5 m long multi-optical path for Fourier-transform infrared spectroscopy (FT-IR) for gaseous composition analysis. The detailed quantification procedure was previously described (Kimura et al. 2019).

### Supply of N_2_O_5_ to plants via liquid medium

Plants were grown with +N medium for 1 week, then pots were transferred to –N medium and grown for another 2 weeks (= nitrogen-deficient plants). To supply N_2_O_5_, 50 ml of –N medium per plant was placed in a container, and N_2_O_5_ gas was supplied for 1 (ca. 2 L), 4 (ca. 8 L) or 7 min (ca. 14 L). The plants with their pots were then transferred to this medium. The N_2_O_5_-dissolved medium was supplied every 3–4 days for a total of three times. The plants were grown for an additional 2 weeks after the last supply and then sampled. As a positive control, +N medium was supplied to nitrogen-deficient plants and, as a negative control, –N medium was supplied to nitrogen-deficient plants.

### Supply of N_2_O_5_ gas to plants

Plants were grown on +N medium for 1 week, transferred to –N medium and then grown for another 2 weeks (= nitrogen-deficient plants). At that point, plants were placed with their pots into secondary containers, and exposed to N_2_O_5_ gas directly at approximately 300 ppm at 2 L/min for 1, 4, or 7 min every 3–4 days for a total of three times. After the final treatment, plants were grown for another 2 weeks and then sampled. For the mock treatment, dry air was supplied for 7 min to nitrogen-deficient plants.

### Supply of N_2_O_5_ and HNO_3_ to plants

Plants were grown on +N medium for one week, transferred to –N medium and grown for 2 weeks more. The plants were then placed into secondary, conical containers and supplied with N_2_O_5_ gas or humidified gas containing HNO_3_ and N_2_O_5_ (see description of plasma gas generation above) for 4 min every 3–4 days for a total of three times. The plants were grown for another 2 weeks after the final treatment and before sampling.

### Long-term exposure to a batch volume of N_2_O_5_ gas

Plants grown on –N medium for 2 weeks after sowing were used. N_2_O_5_ and HNO_3_ gases generated by the plasma device were supplied for 20 min into a 35 L container containing several 0.125 L boxes. A plant was then placed into each 0.125 L box, the box was quickly covered with a lid and placed in a growth chamber for 24 h. The lid was then opened, and the plants remained in the growth chamber, supplied with –N liquid medium until the next treatment. This gas treatment was repeated twice per week, for a total of six treatments. After the final treatment, plants were grown for one more week with –N medium before sampling.

## Results

### N_2_O_5_ is an effective nitrogen source for ***Arabidopsis thaliana***

**Fig. 1:**
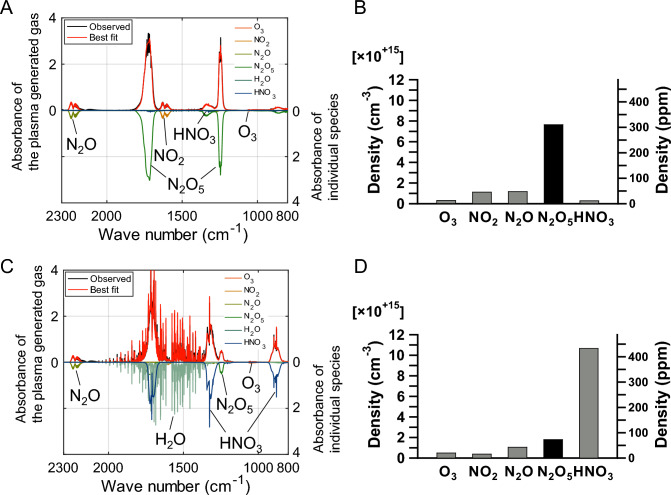
Gaseous composition of plasma-generated N_2_O_5_ gas and HNO_3_ gas. **A**, **B** N_2_O_5_ gas was generated from extremely dry air using an atmospheric pressure plasma device, developed recently (Sasaki et al. 2021). **C**, **D** HNO_3_ gas was generated by mixing humidified air with the extremely dry N_2_O_5_ gas. **A**, **C** A typical Fourier-transform infrared spectroscopy (FT-IR) absorbance spectrum of the plasma-generated N_2_O_5_ gas and HNO_3_ gas (upper half). The best-fit spectrum was obtained with the least square error method. The best-fit spectrum was the summation of the absorbance spectra of the individual species, as shown by the downward absorbance proportional to each density (lower half). **B**, **D** Typical densities of individual gaseous reactive species in dry N_2_O_5_ gas B and in humidified HNO_3_ gas obtained from FT-IR spectra in A and C, respectively.

We employed an atmospheric pressure plasma portable device, which we recently developed, to selectively generate N_2_O_5_ gas from ambient air with low power consumption (< 100 W) (Sasaki et al. 2021). The composition of the generated gas was highly dependent on the humidity of the air used as substrate. When dry air was used with the device, most of the generated gas consisted of N_2_O_5_ (Fig. 1A, B). The resulting dry gas contained 311 ppm of N_2_O_5_. When the dry N_2_O_5_ containing air was mixed with humidified air, the N_2_O_5_ gas immediately converted to HNO_3_, and the main product was HNO_3_ (Fig. 1C, D). The resulting humidified gas contained 433 ppm of HNO_3_ and 73 ppm of N_2_O_5_.

**Fig. 2:**
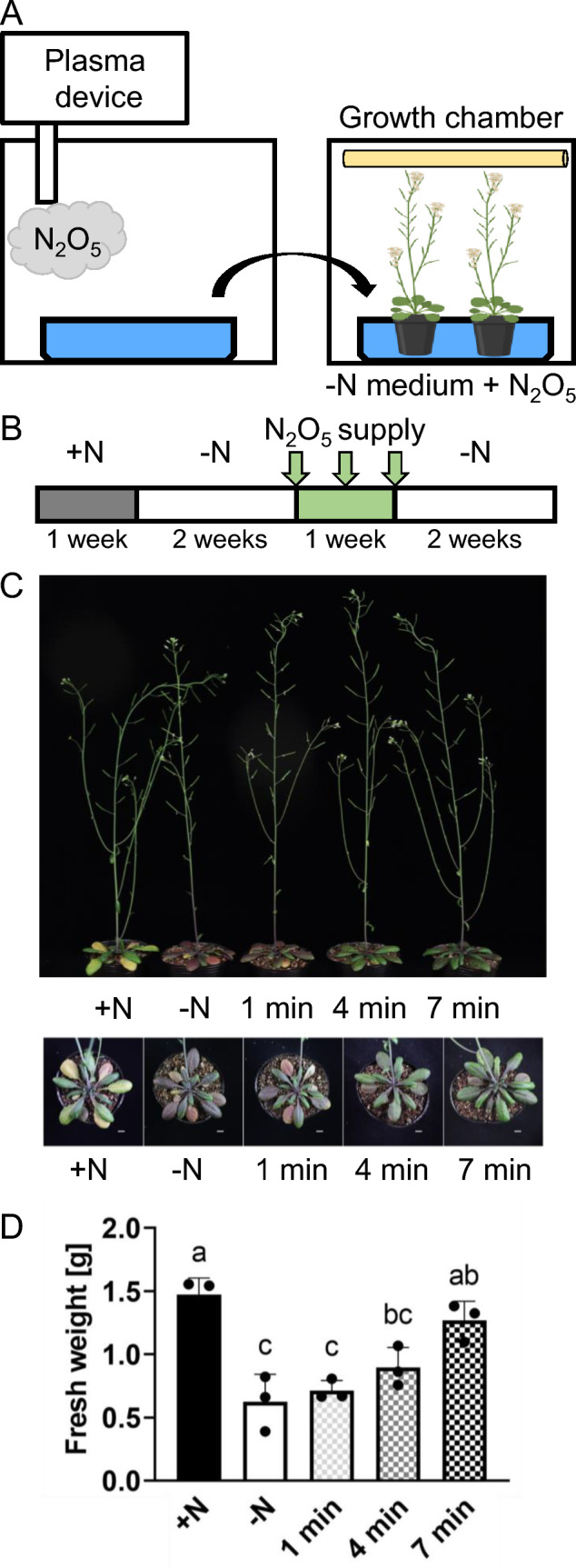
Plasma-generated N_2_O_5_ is an effective nitrogen source for plants. **A** Experimental set-up for the treatment of plants with N_2_O_5_ and the following plant culture. **B** Plants were initially grown with a nitrogen-sufficient medium containing Ca(NO_3_)_2_ (+N) for a week (gray box) and then shifted to nitrogen-deficient medium (N) for 2 weeks (white box). After that, plants were supplied with medium treated with N_2_O_5_ gas (for 1, 4 or 7 min, see Methods for details) every 3–4 days for a total of three times (green box). Following these treatments, the plants were grown on N medium for two more weeks (white box) before being photographed and sampled for phenotype analysis. For further details refer to Methods and Supplemental Table S1. **C** Photograph of plants taken at the end of the experiment. **D** Fresh weight of the plants. This experiment was repeated twice. The data are presented as mean ± s.d. of n ≥ 3 plants, analyzed using one-way ANOVA

To evaluate the potential of N_2_O_5_ gas to be used as a nitrogen source for plant growth, we applied the generated N_2_O_5_ gas to nitrogen-free medium for 1, 4 or 7 min and used this medium to treat nitrogen-deficient plants (Fig. 2A). Specifically, after culturing plants with nitrogen-sufficient medium for 1 week, they were cultured with nitrogen-free medium for 2 weeks to induce nitrogen-deficiency (Fig. 2B and Supplemental Table S1). Then, the plants in the pots were supplied with medium that had been exposed to N_2_O_5_ gas (for 1, 4 or 7 min). Plants remained in this medium for 1 week and were then transferred back to medium without nitrogen source. Growth of plants treated with medium exposed to N_2_O_5_ gas for 1 or 4 min was similar to that of plants grown under nitrogen-deficient conditions (Fig. 2C, D). However, plants treated with medium exposed to N_2_O_5_ gas for 7 min grew as well as those grown with nitrogen-sufficient medium that contained Ca(NO_3_)_2_ (Fig. 2C, D). Furthermore, accumulation of anthocyanins, a symptom of nitrogen deficiency, decreased the longer the medium had been exposed to N_2_O_5_ (Fig. 2C). This suggested that plasma-generated N_2_O_5_ gas can be used as a nitrogen source for plant growth.

### Treating plants directly with plasma-generated N_2_O_5_ gas has negative effects on growth

**Fig. 3:**
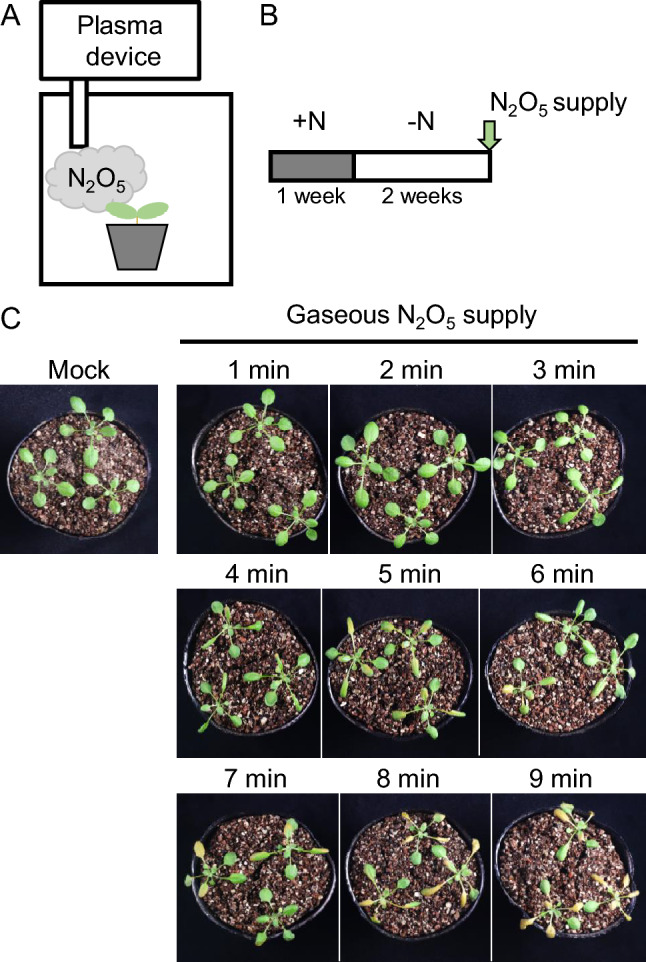
Effects of direct supply of N_2_O_5_ gas to plant seedlings. **A** Experimental set-up for the treatment of plants with N_2_O_5_ and the following plant culture. **B** Plants grown for 1 week on +N medium were transferred to –N medium and grown for another 2 weeks. Then N_2_O_5_ gas was directly supplied to each plant for 1 to 9 min. Mock treatment consisted of dry air supplied to the plants for 9 min. **C** Photographs of plants, taken after one treatment

We next investigated whether N_2_O_5_ gas could be used to promote plant growth by directly supplying it to the plants as a gas. Considering the quick conversion of N_2_O_5_ gas to NO_3_^−^ in water (Fig. 1), we predicted that N_2_O_5_ gas might be readily absorbed by the vermiculite and plant tissues (leaves and stems). N_2_O_5_ gas generated by the same portable plasma device was applied to plants over a short period of time (1–9 min). Under those conditions, symptoms of plant damage were observed after about 4 min of treatment, and significant changes in the plants were evident after 7 min of the treatment (Fig. 3B). Plants exposed to the gas for 7 min began to wilt and change their leaf color within minutes (Fig. 3B and Supplemental Fig. S1). Therefore, the direct supply N_2_O_5_ through the portable plasma device had negative impacts on plant health.

**Fig. 4:**
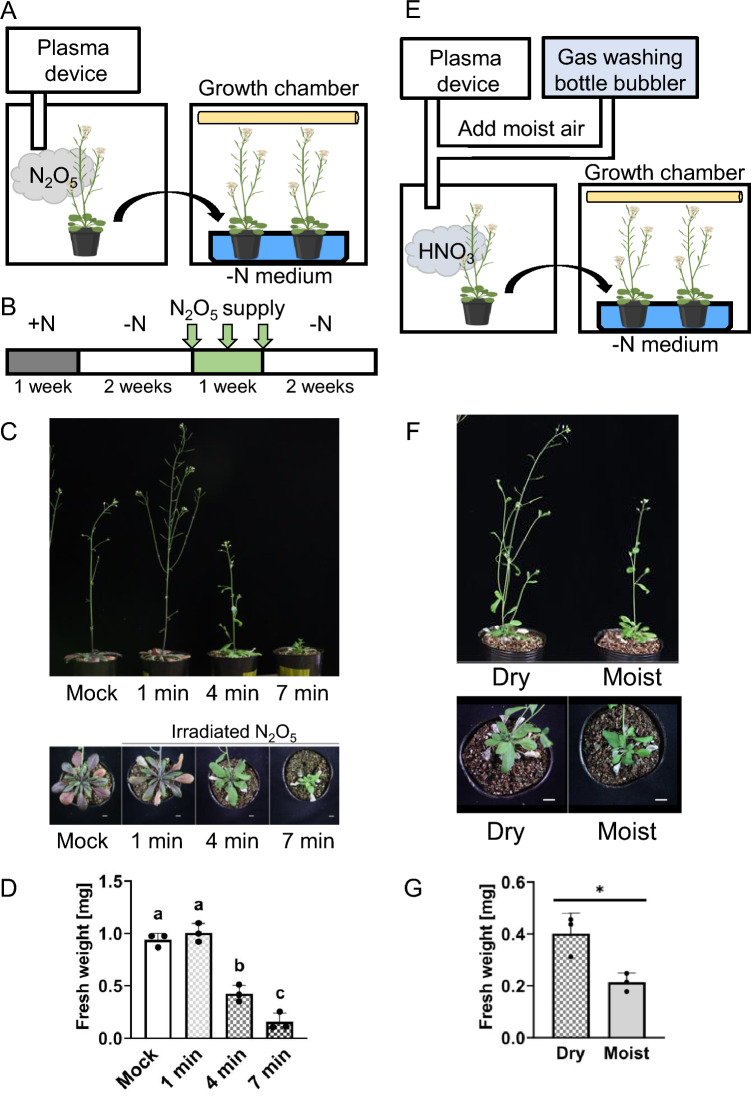
Direct exposure to plasma-generated N_2_O_5_ gas impaired plant growth. **A** Experimental set-up for the treatment of plants with N_2_O_5_ and the following plant culture. **B** Plants were grown on +N medium for 1 week (grey box), followed by growth on –N medium for two weeks (white box), and then treated with dry air (mock) or with N_2_O_5_ gas added directly to the vapor phase of the container. The gas was injected for 1, 4, or 7 min each time, every 3–4 days for a total of three times (green box). After the final treatment, the plants remained on –N medium for two more weeks (white box) before being photographed and sampled for phenotype analysis. **C** Photographs of plants taken at the end of the experiment. **D** Fresh weight of the plants. This experiment was repeated twice. The data are presented as mean ± s.d. of n = 3 plants, analyzed using one-way ANOVA. **E** Experimental set-up for the treatment of plants with dry or humidified N_2_O_5_ gas and the following plant culture, providing a simplified method of supplying N_2_O_5_ and HNO_3_ to plants. The experimental schedule was essentially the same as B except for the N_2_O_5_ treatment. Three-week-old plants were either exposed to generated N_2_O_5_ directly or to N_2_O_5_ that had been additionally passed through humid air for 4 min. The treatment was performed every 3–4 days for a total of three times before plants were photographed and sampled for phenotype analysis. **F** Photographs of plants taken at the end of the experiment. **G** Fresh weight of the plants. This experiment was repeated twice. The data are presented as mean ± s.d. of n = 3 independent plants P < 0.05,* ns* not significant (P > 0.05); Student t-test. Scale bars = 1 cm

To supply a sufficient amount of nitrogen to the plants without prolonged direct exposure to N_2_O_5_ gas, we instead supplied N_2_O_5_ gas to the plants for a limited period of 1, 4, or 7 min at three separate times, 3–4 days apart (Fig. 4A, B and Supplemental Table S1). Reproductive-stage plants grown under nitrogen-deficient conditions were used for this experiment. Leave damage was observed in plants exposed to N_2_O_5_ gas for 4 min and 7 min, and fresh weight was reduced by 71% and 89% when plants were treated three times for 4 min and 7 min, respectively, compared to the mock-treated plants (Fig. 4C, D). However, plants exposed to N_2_O_5_ gas three times for 4 or 7 min had green leaves, and symptoms of nitrate deficiency disappeared (Fig. 4C). Plants exposed to N_2_O_5_ three times for 1 min showed accumulation of anthocyanin in their leaves although their fresh weight was almost the same as that of the mock-treated plants.

We evaluated the effects of mixing N_2_O_5_ gas with moist air on plant growth (Fig. 4e) because under high humidity N_2_O_5_ was converted to HNO_3_ gas (Fig. 1). Air was pre-saturated with humidity by flowing through a Gas washing bottle bubbler, and then mixed with N_2_O_5_ gas (Fig. 4E). The resulting gas mixture was added to the plant growth chamber every 3–4 days for 4 min, a total of three times (Fig. 4B, Table S1). Control treatment with N_2_O_5_ mixed with dry air was done the same as shown in Fig. 2. When plants were exposed to N_2_O_5_ gas mixed with moist air, they produced less biomass but continued to look healthy without chlorosis (Fig. 4F). Plants treated with dry N_2_O_5_ gas had an 88% increase in fresh weight, compared with plants treated with the humidified N_2_O_5_ gas (Fig. 4G). These data suggested that treating plants with dry N_2_O_5_ gas was better for plant growth than treatment with N_2_O_5_ gas mixed with moist air.

### Alternative modes of application of N_2_O_5_ to plants

**Fig. 5:**
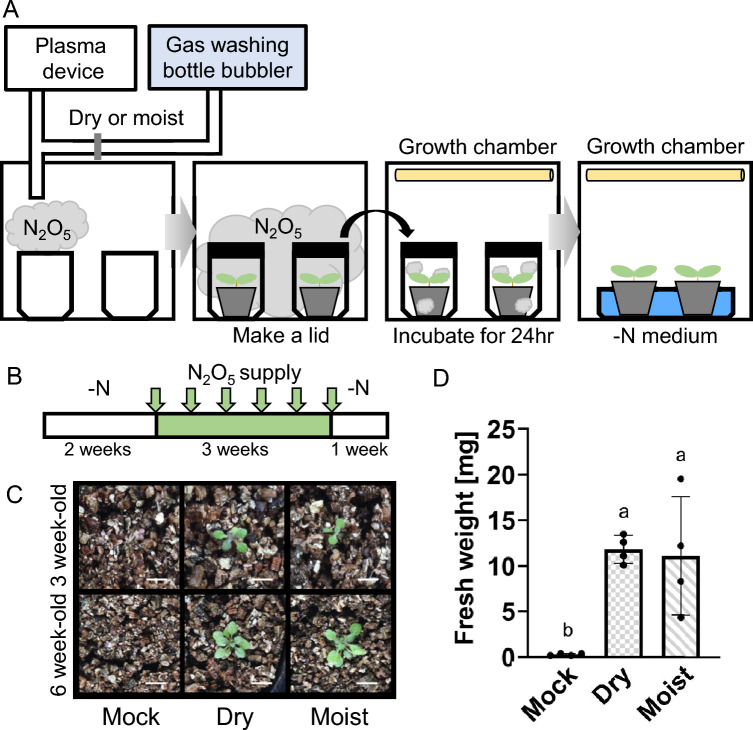
Supplementation of generated N_2_O_5_ to plants without direct exposure to N_2_O_5_ gas. **A** Experimental set-up for the treatment of plants with N_2_O_5_ and the following plant culture. **B** After germinating seeds on –N medium and growing them for 2 weeks (white box), the pots were placed in containers filled with plasma-generated N_2_O_5_ or HNO_3_ gas generated according to the process shown in Fig. 1B and containers remained sealed for one day. After that, the plant pots were removed from the containers and placed directly into the growth chamber for 2 or 3 days. This treatment cycle was repeated five times for a total of 6 treatments over the course of 3 weeks (green box, arrowheads indicate treatments). After the final treatment, plants were grown for another one week in a growth chamber (white box) before being photographed and sampled for phenotype analysis. Further details refer to Materials and Methods and Supplemental TableS2 . **C** Photographs of plants at the end of the experiment. Scale bars = 1 cm. **D** Fresh weight of the plants. This experiment was repeated twice. The data are presented as mean ± s.d. of n = 4 plants, analyzed using one-way ANOVA

The direct supply of N_2_O_5_ gas as shown in Figs. 3 and 4 inhibited the growth of plants. Therefore, we tested an alternative method of supplying nitrogen directly from the N_2_O_5_ generator to plants in nitrogen-free medium. For this, we physically separated the plant culture process from the treatment with N_2_O_5_ (Fig. 5A, B). First, N_2_O_5_ gas generated by the device (either mixed with dry or moist air) was introduced for 20 min into a container holding several smaller containers but no plants. After that, nitrogen-deficient plants were placed into the smaller containers which were covered with lids. Note that this process was feasible since N_2_O_5_ gas has a higher density than air. The covered containers with the plants were moved to a growth chamber. After 24 h, the lids were removed, and plants were again supplied with nitrogen-free medium (Fig. 5A). This procedure was repeated twice per week, for a total of six times, followed by an additional week of growth with nitrogen-free medium at the end (Fig. 5B and Supplemental Table S2). In this result, plants treated with dry air could hardly grow on vermiculite and this suggested vermiculite did not have sufficient amount of nitrogen for plant growth. Plants treated with N_2_O_5_ gas had increased fresh weight, similar to the plants treated with HNO_3_ gas (resulting from mixing N_2_O_5_ gas with moist air) (Fig. 5C, D). These results showed that supplying N_2_O_5_ gas to plants can be an effective method for supplying nitrogen fertilizers to plants without relying on traditional nitrogen fixation processes.

## Discussion

N_2_ in the air is an inert gas that is not readily available as a plant nutrient but instead requires conversion by energy-intensive conventional nitrogen fixation processes. Our study shows that N_2_O_5_ gas generated with a portable plasma device can be a new avenue of supplying a nitrogen source to plants. This method can be expected to be useful as an alternative nitrogen fertilization procedure to support plant growth while preventing the excessive use of conventional nitrogen fertilizers. However, direct administration of N_2_O_5_ to plants inhibited growth (Fig. 3). Hence, it was crucial for us to develop an effective method for delivering N_2_O_5_ to the plants. In this study, we explored various methods for administering N_2_O_5_ gas to plants. When plants were cultured using liquid medium that had been exposed to N_2_O_5_ gas, their growth was not impaired (Fig. 2). But repeated short-term application of the N_2_O_5_ gas directly from the plasma device to nitrogen-starved plants damaged shoot tissues and only partially improved plant growth (Fig. 4). These problems could be avoided by placing nitrogen-deficient plants into a chamber pre-filled with N_2_O_5_, and subsequently growing them in medium without a nitrogen source (Fig. 5). Our results demonstrated that plants could be grown from seeds on nitrogen-free medium, with nitrogen supplied from the air, in the form of N_2_O_5_ gas if the duration of contact with the plants or the concentration of the gas was reduced.

The portable plasma device used in this study consumes less than 100 W of power, which can be efficiently supplied from renewable energy sources, and the only material used is air. This device excels in its portability and ability to produce a nitrogen source from air using minimal power, without the need for large facilities. The device is an efficient N_2_O_5_ generator that can selectively produce N_2_O_5_, minimizing waste (Fig. 1) (Sasaki et al. 2021). On the other hand, the energy efficiency of nitrogen fixation by the plasma device is about 72 MJ/(mol N) (Sasaki et al. 2021), and that by the Haber–Bosch method is about 0.5 to 0.6 MJ/(mol N) (Rouwenhorst et al. 2021), indicating that the Haber–Bosch method is still superior to the portable plasma device in terms of energy efficiency per mole of nitrogen.

The generated N_2_O_5_ gas contained some impurities including O_3_ (Fig. 1), which is known to cause cell death and photosynthesis inhibition in Arabidopsis even at very low concentrations at the ppb level. As shown in Fig. 3 and 4, direct exposure of plants to N_2_O_5_ gas produced by the device resulted in growth impairment, potentially due to the presence of O_3_ or rapid acidification due to the chemical reaction of N_2_O_5_ and H_2_O. However, when the supply of plasma-generated N_2_O_5_ gas was more limited, the plants grew well (Fig. 5).

Foliar application of liquid fertilizers is used in modern agriculture. This technique is especially effective in plants with weakened roots and under continuous cloud cover (high humidity) where transpiration does not take place (Fernández et al. 2021; Naqve et al. 2021). Therefore, foliar application allows nitrogen to be supplied directly to the tissues that need it. Foliar nitrogen is supplied as water-soluble compounds including NO_3_^−^, NH_4_^+^, urea and amino acids (Lv et al. 2021). This method has the disadvantage of not supplying nitrogen uniformly due to high leaf overlap and plant density. In contrast, gaseous nitrogen fertilizers can even penetrate into small spaces between leaves. The chemical polarity of N_2_O_5_ is significantly lower than that of HNO_3_. This lower polarity may promote the infiltration of N_2_O_5_ across the leaf surface, consisting of the cuticular wax and cell wall layers. Once taken up into the cells, the buffering capacity of the cytoplasm may ameliorate any acidification resulting from the conversion of N_2_O_5_ to HNO_3_. This may explain why treatment with dry N_2_O_5_ was more effective in enhancing plant growth than when N_2_O_5_ was mixed with moist air (as shown in Fig. 4F, G). Moreover, it was recently reported that exposure of *Arabidopsis thaliana* to N_2_O_5_ gas leads to the induction of an immune response during a pathogen inoculation test (Tsukidate et al. 2022). This indicates that N_2_O_5_ is absorbed by the surface of the plant tissue. The immune-inducing effect of N_2_O_5_ also increases its superiority for use as a nitrogen fertilizer for plants. Our study supports the idea that air-derived N_2_O_5_ has potential to be used as a foliar nitrogen fertilizer altered to or with conventional nitrogen fertilizer although N_2_O_5_ was also absorbed by the soil and the medium.

In conclusion, we have successfully applied N_2_O_5_ gas as alternative nitrogen source that improves plant growth. This ability to directly utilize N_2_, which is abundantly present in the Earth’s atmosphere, as a gaseous nitrogen source is a promising development for plant cultivation and agriculture. The method established in this study will even make it feasible to reliably supply nitrogen to regions of the world where nitrogen fertilizer production has been challenging or impossible, such as in less developed regions.

### Supplementary Information

Below is the link to the electronic supplementary material.

Supplementary file1 (PPTX 17369 kb)

## Data Availability

This ability to directly utilize N2, which is abundantly present in the Earth’s atmosphere, as a gaseous nitrogen source “may be” a promising development for plant cultivation and agriculture.
