# Supplementary material for: Utilizing plasma-generated N2O5 gas from atmospheric air as a novel gaseous nitrogen source for plants
Source: Plant Mol Biol. 2024 Apr 8;114(2):35. doi: 10.1007/s11103-024-01438-9 (PMC11001677; doi:10.1007/s11103-024-01438-9)
Supplement: Supplementary file 1 — Supplementary file1 (PPTX 17369 kb) [file 11103_2024_1438_MOESM1_ESM.pptx]

## Slide 1
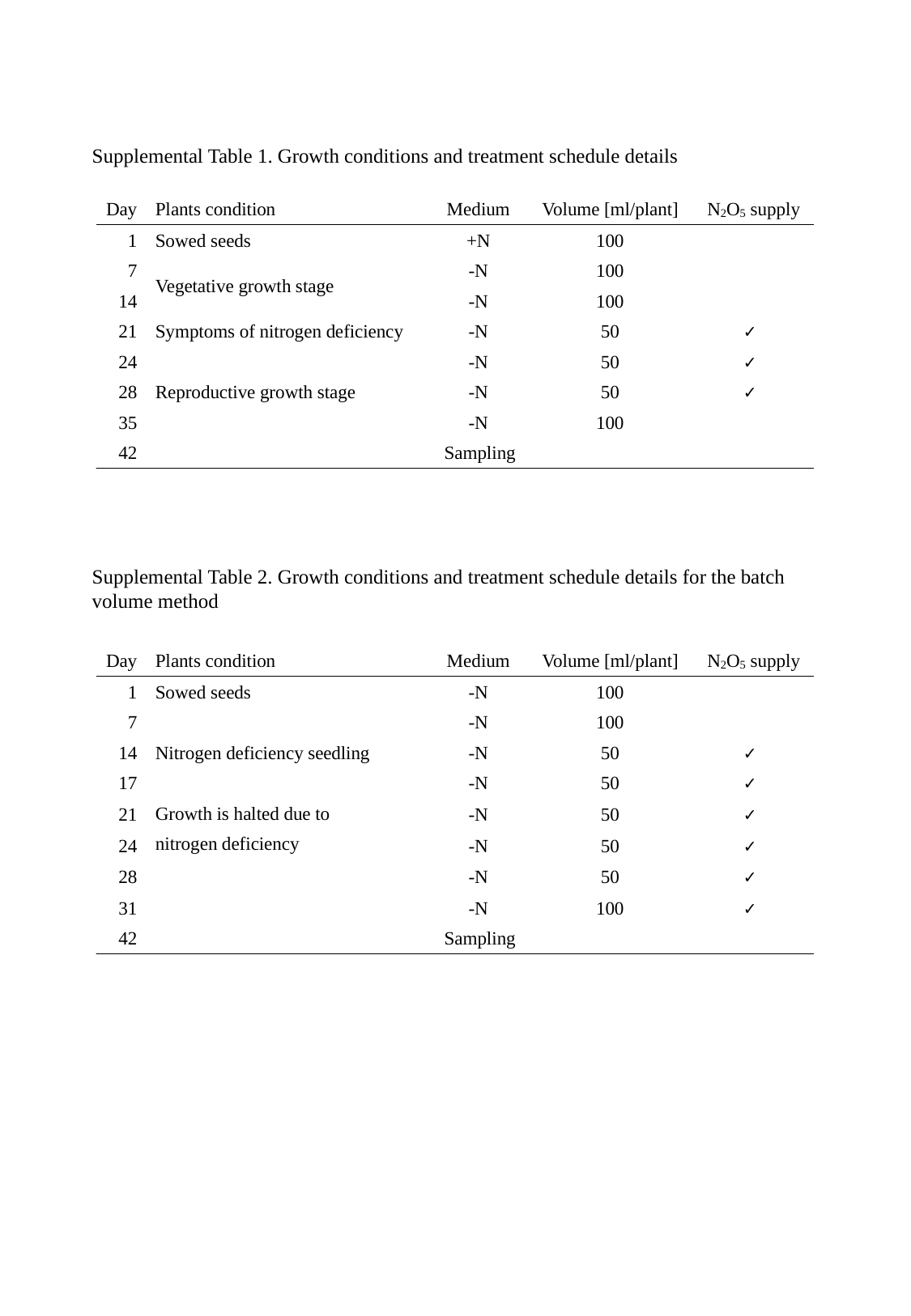

Supplemental Table 1. Growth conditions and treatment schedule details
Supplemental Table 2. Growth conditions and treatment schedule details for the batch volume method

## Slide 2
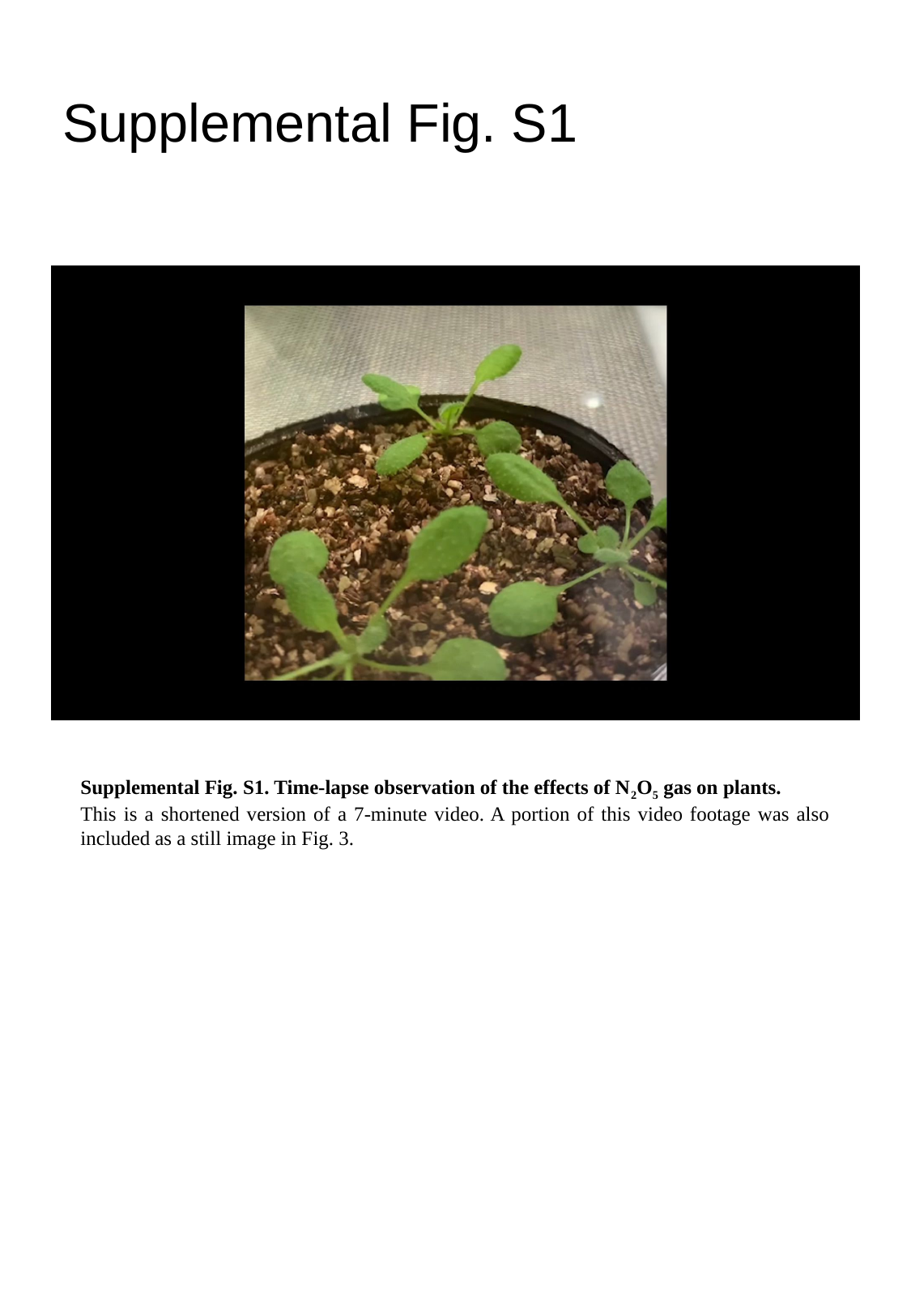

Supplemental Fig. S1
Supplemental Fig. S1. Time-lapse observation of the effects of N2O5 gas on plants.
This is a shortened version of a 7-minute video. A portion of this video footage was also included as a still image in Fig. 3.

## Slide 3
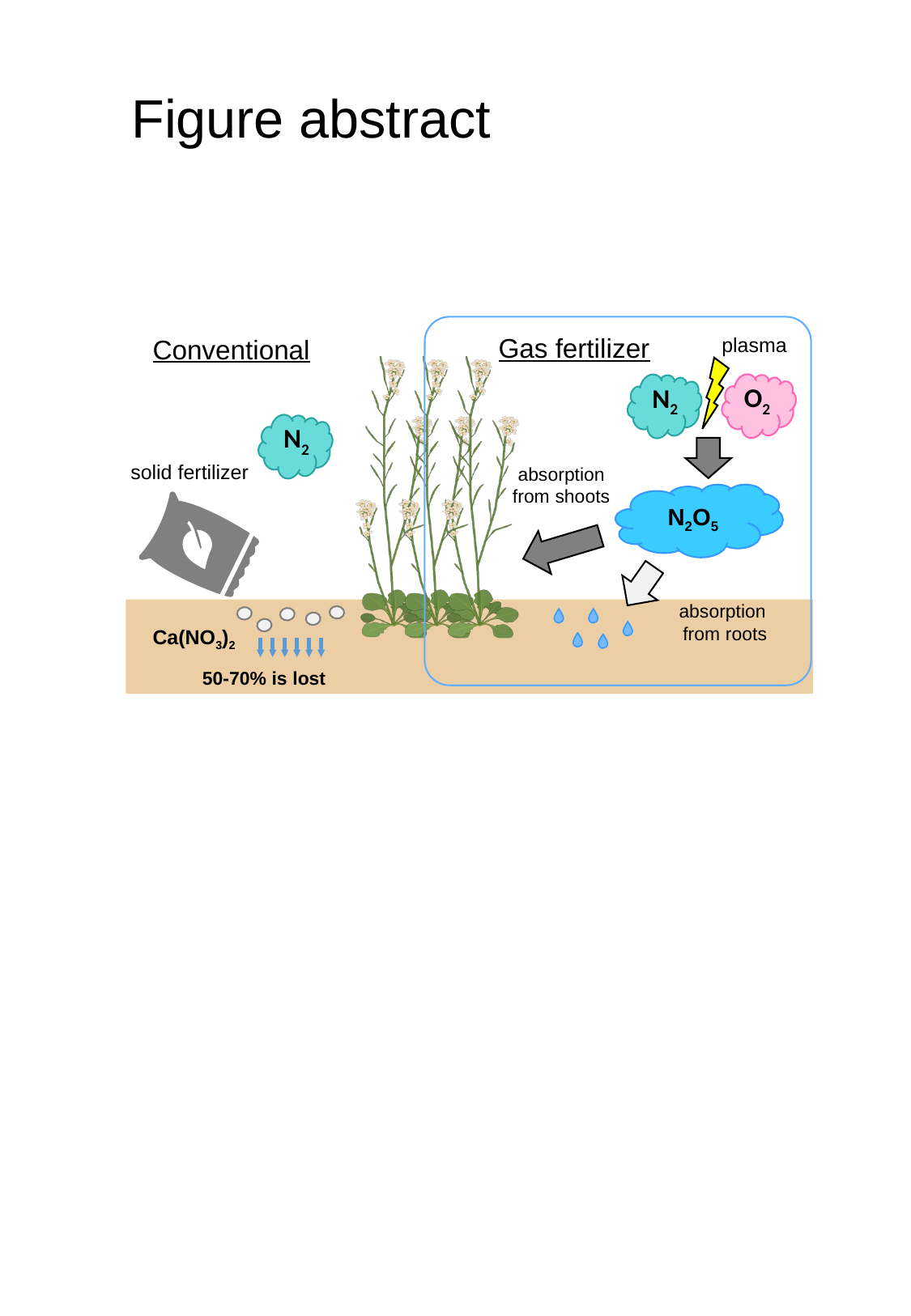

Figure abstract
Gas fertilizer
plasma
Conventional
O2
N2
N2
solid fertilizer
absorption
from shoots
N2O5
absorption
from roots
Ca(NO3)2
50-70% is lost
